# Reliable cell and tissue morphology-based diagnosis of endemic Burkitt lymphoma in resource-constrained settings in Ghana

**DOI:** 10.1186/s12885-019-6488-1

**Published:** 2019-12-30

**Authors:** Cecilia Smith-Togobo, Mette Ø. Pedersen, Steffen G. Jensen, Babatunde Duduyemi, Richard K. Gyasi, Michael F. Ofori, Vivian Paintsil, Lorna Renner, Peter Nørgaard, Lars Hviid

**Affiliations:** 1 Department of Biochemistry, Cell and Molecular Biology, University of Ghana, Legon, Ghana; 2 Department of Immunology, Noguchi Memorial Institute for Medical Research, University of Ghana, Legon, Ghana; 3 Centre for Medical Parasitology at Department of Immunology and Microbiology, Faculty of Health and Medical Sciences, University of Copenhagen, Copenhagen, Denmark; 4 Department of Pathology, Herlev and Gentofte Hospital, Herlev, Denmark; 5 Department of Pathology, Komfo Anokye Hospital, Kumasi, Ghana; 6 Department of Pathology, Korle-Bu Teaching Hospital, Accra, Ghana; 7 Department of Child Health, Komfo Anokye Hospital, Kumasi, Ghana; 8 Department of Child Health, Korle-Bu Teaching Hospital, Accra, Ghana; 9 Department of Infectious Diseases, Rigshospitalet, Copenhagen, Denmark

**Keywords:** Endemic Burkitt lymphoma, Diagnostic accuracy, Morphology, C-MYC immunohistochemistry, *c-myc* FISH

## Abstract

**Background:**

Endemic Burkitt lymphoma (eBL) is an aggressive B-cell lymphoma, which is a common childhood cancer in areas with intense transmission of *Plasmodium falciparum* parasites. Early and accurate diagnosis is a prerequisite for successful therapy, but it optimally involves advanced laboratory investigations. These are technologically demanding, expensive, and often difficult to implement in settings where eBL is prevalent. Diagnosis is thus generally based on clinical assessment and morphological examination of tumour biopsies or fine-needle aspirates (FNAs).

**Methods:**

The purpose of the present study was to assess the accuracy of eBL diagnosis at two tertiary hospitals in Ghana. To that end, we studied FNAs from 29 eBL patients and 21 non-eBL lymphoma patients originally diagnosed in 2018. In addition, we examined 111 archival formalin-fixed and paraffin-embedded (FFPE) biopsies from Ghanaian patients originally diagnosed as eBL (*N* = 55) or non-eBL (*N* = 56) between 2010 and 2017. Availability-based subsets of samples were subjected to haematoxylin-eosin or Giemsa staining, C-MYC immunohistochemistry, and fluorescence in situ hybridisation (FISH) analysis of *c-myc* rearrangements.

**Results:**

We found a good correlation between original diagnosis and subsequent retrospective assessment, particularly for FNA samples. However, evidence of intact *c-myc* genes and normal C-MYC expression in samples from some patients originally diagnosed as eBL indicates that morphological assessment alone can lead to eBL over-diagnosis in our study area. In addition, several FFPE samples could not be assessed retrospectively, due to poor sample quality. Therefore, the simpler FNA method of obtaining tumour material is preferable, particularly when careful processing of biopsy specimens cannot be guaranteed.

**Conclusion:**

We conclude that the accuracy of eBL diagnostic tools available in Ghana is generally adequate, but could be improved by implementation of additional pathology laboratory investigations. Improved attention to adequate preservation of archival samples is recommended.

## Background

Endemic Burkitt lymphoma (eBL) is an aggressive B-cell lymphoma (ABCL) [1], first described by the British surgeon Dennis Burkitt [2]. The disease is a common childhood cancer in areas of sub-Saharan Africa, where transmission of the malaria parasite *P. falciparum* is intense [3, 4]. In Ghana, eBL has been the most common cancer and cause of cancer death among children for decades [5, 6]. Diagnosis of eBL in Ghana, as in many low-income countries, is based on clinical presentation, ultrasound scans (when available), and assessment of tumour cell/tissue morphology in FNAs or biopsies. As other B-cell lymphomas can have similar morphological features to eBL, it is prudent to strengthen morphological diagnosis by specific immunohistochemistry and/or by fluorescence in situ hybridization (FISH) [7]. However, this is rarely possible in a resource-constrained setting [8]. While previous studies have indicated high concordance between clinical and molecular diagnosis of a range of ABCLs, they were done outside areas endemic for eBL and thus did not include eBL patients [9]. Diagnostic accuracy is likely to be lower in resource- constrained settings [10]. We therefore set out to analyse to what extent morphology-based diagnosis of eBL in Ghana could retrospectively be confirmed morphologically and molecularly by immunohistochemistry and FISH. Samples from paediatric patients admitted in 2018 to the two major referral hospitals in the country and diagnosed with eBL or non-eBL, as well as archival (2009–2017) formalin-fixed and paraffin-embedded (FFPE) tissue samples from eBL and non-eBL patients were included in the study.

## Methods

### Ethical issues

The study protocol was approved by the institutional review boards of Kwame Nkrumah University of Science and Technology (CHRPE/AP/175/17) and Korle-Bu Teaching Hospital (KBTH; IRB/00080/2016) and by the Heads of Department of the pathology units of Komfo Anokye Teaching Hospital (KATH) and KBTH. Written informed consent was obtained from the parents/guardians on admission before inclusion of patients in the study.

### Participants and samples

**Fig. 1:**
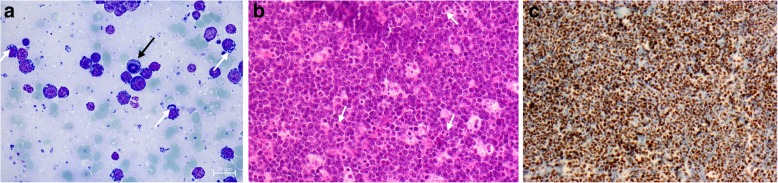
Morphology of Burkitt lymphoma. Giemsa-stained FNA smear from an eBL tumour with cells showing characteristic cytoplasmic vacuoles (white arrows) and a cell with a prominent mitotic figure (black arrow) (**a**). Haematoxylin-eosin-stained FFPE tumour tissue section (20× magnification) showing cells with mitotic figures (arrows) and characteristic “starry sky” staining due to weakly stained macrophages among numerous densely stained tumour cells (**b**). Immunohistochemistry-stained FFPE tumour tissue section showing prominent C-MYC expression (brown) (**c**)

**Fig. 2:**
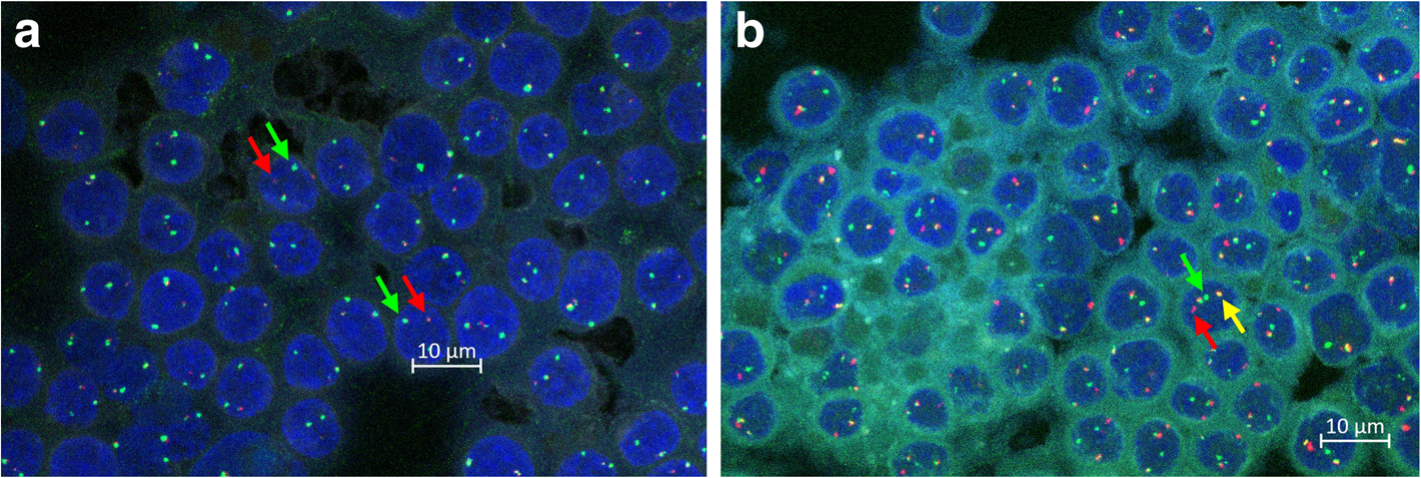
FISH micrographs *c-myc* split probe labelling of the upstream (green) and downstream (red) part of *c-myc* gene in an FNA smear from an eBL patient (separation of red and green fluorescence (red and green arrows) indicating *c-myc* breakage (**a**). *c-myc-igh* fusion probe labelling of *c-myc* (green) and *igh* (red) in an FNA smear from an eBL patient (co-localization (yellow) demonstrates translocation of *c-myc* to *igh*) (**b**)

Patients (*N* = 50) with head, neck, or abdominal masses suggestive of eBL were recruited into the study in 2018 according to the recommendation of the attending physicians (Table 1). Tumour FNAs were obtained at admission for preparation of smears for diagnostic purposes. Additional smears were stored at 4 °C for retrospective and independent assessment of cell morphology (Fig. 1a) and cytogenetics (Fig. 2).

**Table 1:** Characteristics of patients included in the study

			Number	Age (years)	Sex (F/M)	Tumour location (Head/Abd/other)
Study cohort (2018)	eBL	KBTH	7	6.6 (2-12)	4/3	3/4/0
		KATH	22	7.9 (3-13)	6/16	9/13/0
		All	29	7.6 (2-13)	10/19	12/17/0
	Non-eBL	KBTH	8	6.1 (1-13)	3/5	3/5/0
		KATH	13	8.5 (3-15)	7/6	8/5/0
		All	21	7.6 (1-15)	10/11	11/10/0
Archival samples (2009-2017)	eBL	KBTH	35	9.8 (2-25)	11/24	11/18/6
		KATH	20	8.1 (1-15)	7/13	11/9/0
		All	55	9.2 (1-25)	18/37	22/27/6
	Non-eBL	KBTH	37	9.6 (2-15)	8/29	25/6/6
		KATH	19	10.0 (6-15)	2/17	17/2/0
		All	56	9.8 (2-15)	10/46	42/8/6

Archival FFPE tumour samples (*N* = 111) from patients diagnosed with eBL or other childhood lymphomas between 2010 and 2017 were retrieved (Table 1), and new sections (3–4 μm thickness) were cut. The tissue sections were mounted on pre-coated slides (Leica BOND™ Plus) and stored at − 20 °C for later haematoxylin-eosin staining (Fig. 1b) and additional tests.

Only a limited number of specimen slides were available for retrospective analysis, and complete morphological, immunohistochemical, and FISH characterization was therefore not possible.

### Morphological assessment

Retrospective morphological assessment of tumour FNA smears and FFPE tumour sections was performed by two experienced morphologists (CS, PHN) blinded to the original diagnosis. For FNAs, the diagnosis of eBL was based on finding of monotonous, medium-sized, blastoid cells with basophilic cytoplasm usually containing lipid vacuoles and round nuclei with finely clumped chromatin containing multiple medium-sized basophilic nucleoli. For the FFPE sections, the eBL diagnosis was based on finding of a solid tumour composed of medium-sized, blastoid cells with diffuse monotonous growth pattern, cohesive with squared-off borders of retracted basophilic cytoplasm (usually with visible lipid vacuoles), and round nuclei with finely clumped chromatin containing multiple medium-sized basophilic nucleoli. The eBL tumours also contained many tingible body macrophages, giving rise to a characteristic “starry sky” appearance. Mitotic figures were common due to the high proliferation rate of the tumour cells. Only vital and sufficiently fixated tumour tissue was evaluated, and samples containing solely necrotic tissue and/or autolyzed tumour tissue were discarded.

### FISH staining of tissue sections

The frozen sections were brought to room temperature and fixed to the slides (58 °C, 45 min). Paraffin was removed in three changes of Tissue-Clear (Sakura Finetek Europe B.V.; 10 dips in the first two changes and incubation (15 min) in the third), followed by three changes of 100% ethanol (10 dips in the first two and incubation (1 min) in the last change). The sections were subsequently washed in two changes of 96% ethanol followed by two changes of 70% ethanol (10 dips in the first, and incubation (1 min) in the second change of each). The sections were then rinsed by incubation in two changes (3 min each) of wash buffer (Tris-HCl buffer, pH 7.6), followed by pre-treatment buffer (MES-buffer, pH 6.55) at boiling temperature (10 min) in a microwave oven (Whirlpool, CRISP) and cooling (15 min) in the pre-treatment buffer. The sections were then rinsed in two changes of wash buffer as above, blotted with tissue paper and digested with pepsin (ZytoVision, GmbH, Germany; 8 min, room temp), rinsed again as above, dehydrated in two changes of 70, 96, and 100% ethanol (10 dips in first change of each, and incubation (2 min) in the second). To further enhance dehydration, sections were subsequently air-dried (room temp, 15 min). *c-myc/igh* fusion probes (ZytoVision, Germany, z-2105-200) and *c-myc* dual split probes (DAKO, Agilent, USA) were added (1.5-10 μL, depending on the size of the section), the sections covered with coverslips (12 × 12 or 22 × 22 mm^2^) and sealed with Fixogum (ZytoVision, E-4005-126). Following incubation (85 °C, 5 min followed by 37 °C, overnight) of the slides on a hybridiser (ThermoBrite, Statspin, Abbot Molecular), the seals and coverslips were removed, and the sections rinsed (1 min) in Stringent buffer (SSC Buffer 20x concentrate (Sigma), Triton X-100 (Sigma)). The sections were transferred to a Coplin jar filled two-thirds with Stringent buffer and incubated (64 °C, 10 min) in a water-bath to remove unspecific binding of the probes. Finally, the sections were rinsed in two changes of wash buffer, dehydrated, and mounted in fluorescence mounting medium (10 μL) containing Dapi stain (Veatashield®, Vector Lab. Inc., Burlingame, CA 94010). The above procedures were done in batches of ten sections plus known positive (BL tissue) and negative (reactive lymph node) controls.

### FISH staining of FNA smears

FNA smears were fixed (3.7% formalin, 5 min) and rinsed in two changes of wash buffer (3 min each). The smears were further dehydrated as described above and divided into two halves. Probes as above (10 μL each) were added to one half each. The slides were covered, sealed, hybridized and processed further as described above.

### Fluorescence microscopy

Each FISH slide was independently evaluated for *c-myc* break (Fig. 2a) and *c-myg/igh* fusion (Fig. 2b) by three pathology microscopists blinded to the original diagnosis of the patient, using an Olympus BX61 fluorescence microscope equipped with an attached Zeiss camera. For the split probe, the sample was scored positive if > 10% of the red and green signals were clearly separated (Fig. 2a). For the fusion probes, the sample was scored positive if > 10% of the red and green signals were immediately next to each other or overlapping (yellow signal) (Fig. 2b).

### Immunohistochemistry

Expression of C-MYC was evaluated according to the guidelines for routine diagnostic work-up at the Department of Pathology, Herlev Hospital [11]. FFPE tissue sections were pre-treated (pH = 9.0) on a PT Link pre-treatment module (Dako), including paraffin removal, rehydration, and epitope retrieval, and subsequently stained in a Dako Autostainer Link 48, using EnVision FLEX+ visualization kits (Dako) and monoclonal C-MYC antibody Epitomics; clone y69/EP121, 1:100 dilution EnVision Flex Antibody Diluent). All steps were completed according to the manufacturer’s instructions. The stained sections (Fig. 1c) were evaluated and scored by two experienced haemato-pathologists using a double-headed microscope (Olympus BX51, equipped with a colour view camera and analySIS getIT 5.0 software (Soft Imaging Systems Munster, Germany)). MYC expression was evaluated on full slide sections in hot spot areas and all staining intensities were included as previously described [12].

### Statistical analysis

Sensitivity and specificity, and their confidence intervals, were calculated as described in detail elsewhere [13], using the MedCalc online calculator (https://www.medcalc.org/calc/diagnostic_test.php).

## Results

### Morphology analysis

**Fig. 3:**
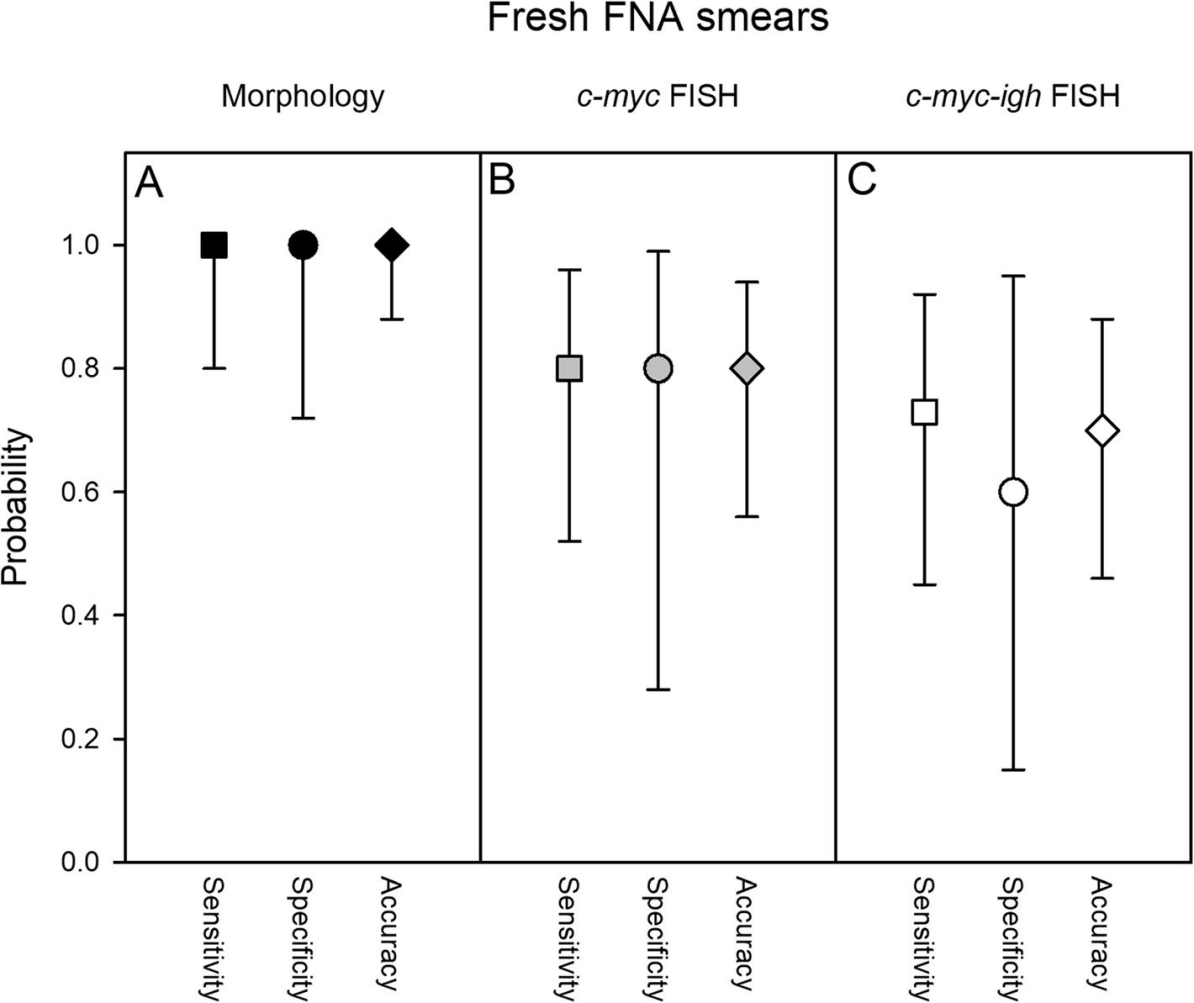
FNA review diagnosis - morphology and FISH. Estimated sensitivity (squares) and specificity (circles), and accuracy (diamonds) of original diagnosis, based on retrospective analysis of cell morphology in Giemsa-stained smears (**a**; black symbols), cytogenetic analysis of *c-myc* breakage (**b**; grey symbols) and *c-myc-igh* translocation (**c**; white symbols) by FISH. Medians (symbols) and 95% confidence intervals (error bars) are shown

Blinded Giemsa-stained FNA tumour smears (Fig. 1a) from 28 of the patients (17 with an original eBL diagnosis) from 2018 (Table 1) were available for retrospective morphological review. The results were in good agreement with the original diagnoses made (Fig. 3a and Additional file 1: Table S1).

**Fig. 4:**
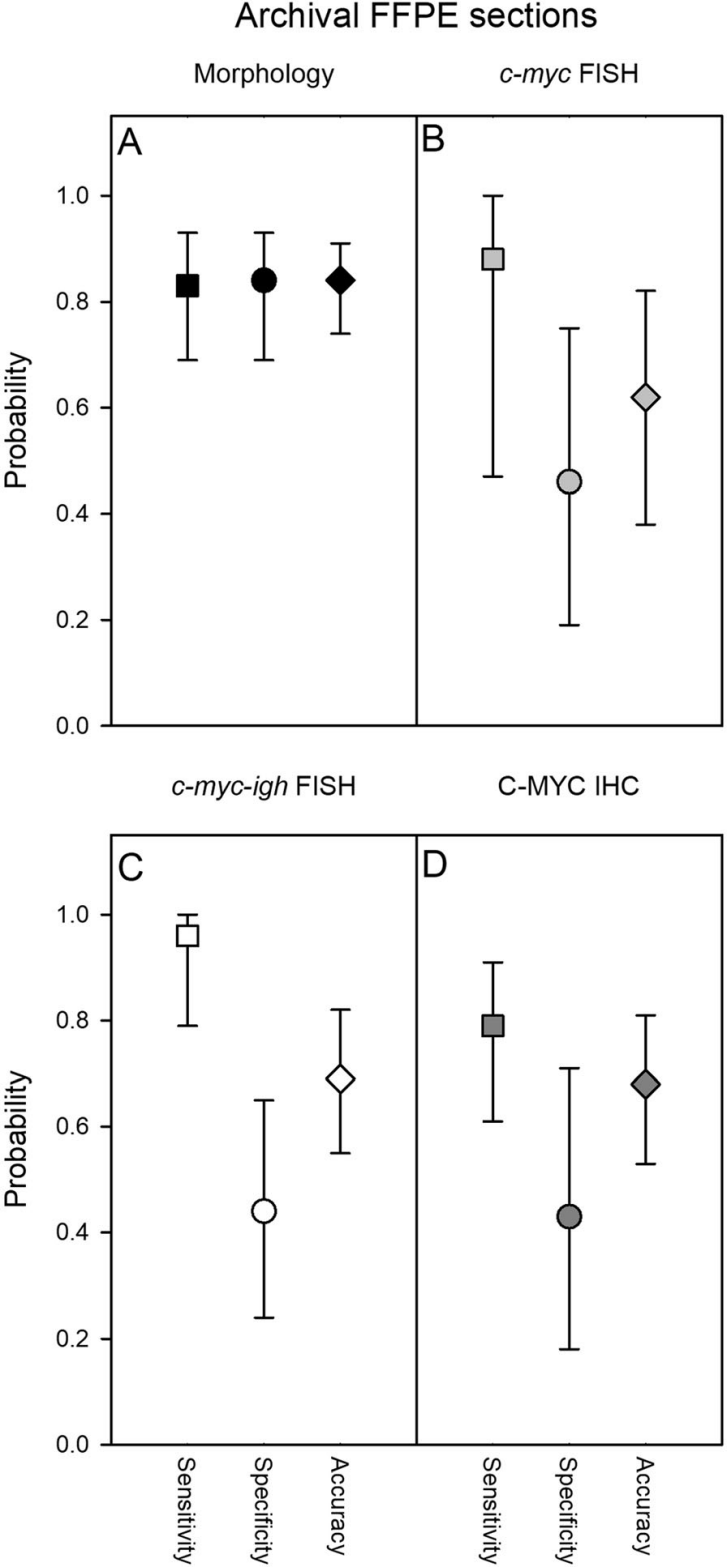
FFPE tissue section review diagnosis – morphology and FISH. Estimated sensitivity (squares), specificity (circles), and accuracy (diamonds) of original diagnosis, based on retrospective analysis of cell morphology in freshly haematoxylin-eosin-stained archival FFPE tissue sections (**a**; black symbols), on cytogenetic analysis of *c-myc* breakage (**b**; grey symbols), *c-myc-igh* translocation (**c**; white symbols) by FISH, or by C-MYC immunohistochemistry (**d**; dark grey symbols) in sections from the archival samples. Medians (symbols) and 95% confidence intervals (error bars) are shown

We next evaluated freshly stained haematoxylin-eosin sections (Fig. 1b) from the archival tissue blocks (Table 1). A reliable retrospective assessment was only possible for 85 (including 42 from patients with an original eBL diagnosis), as the remaining samples had to be discarded because of poor specimen quality (autolysis or no viable tumour tissue due to tumour necrosis) (Additional file 1: Table S2). The overall sensitivity, specificity, and accuracy estimates were somewhat lower than for the FNA smears (Fig. 4a), probably related to the overall lower quality of the archival compared to the fresh samples. The ages of the samples were not significantly related to rejection rate, suggesting that inadequate sample preparation rather than inadequate storage was the main cause of low sample quality.

### Analysis by *c-myc* FISH and C-MYC immunohistochemistry

To further assess the accuracy of the original cell/tissue morphology-based diagnosis, we also analysed FNA smears from 20 of the 2018-patients (including 13 patients originally diagnosed as eBL) for FISH evidence of breakage of the *c-myc* oncogene (Fig. 2a). The sensitivity, specificity, and accuracy estimates (Fig. 3b and Additional file 1: Table S3) were lower than above. We did not find evidence of *c-myc* breakage in one of the patients originally diagnosed as eBL, whereas *c-myc* breakage was detected in four patients originally diagnosed as non-eBL. While *c-myc* breakage is not specific for eBL, lack of *c-myc* breakage is rare in eBL, and this analysis thus suggests some over-diagnosis originally. However, cryptic rearrangements in some cases cannot be formally ruled out [14]. Examination of slides for FISH evidence of *c-myc* translocation into the *igh* locus (Fig. 2b) yielded similar results (Fig. 3c and Additional file 1: Table S4).

Corresponding FISH analysis of archival FFPE sections showed high sensitivity but low specificity (Fig. 4b-c and Additional file 1: Tables S5-S6). The low specificity was due to the fact that we did not find evidence of *c-myc* breakage in half (7/14) of the patients originally diagnosed as eBL. As lack of *c-myc* breakage is rare in eBL, this finding supports the above indication of a degree of over-diagnosis when supportive molecular evidence of eBL is not available. This conclusion is further supported by examination of biopsy sections for immuno-histochemical evidence of C-MYC expression (Fig. 1c). Breakage and translocation of *c-myc* is often associated with high expression of C-MYC protein [15], and immunohistochemistry is generally less sensitive to sample deterioration than FISH [16, 17]. This analysis (Fig. 4d and Additional file 1: Table S7) showed low specificity due to absence of C-MYC expression in biopsies from eight of 34 patients originally diagnosed as eBL.

## Discussion

Endemic Burkitt lymphoma is a highly aggressive extra-nodal tumour of children in areas characterized by early and massive exposure to Epstein-Barr virus and stable transmission of the malaria parasite *P. falciparum* [18, 19]. The disease is therefore largely restricted to equatorial Africa, where it is often the most common paediatric malignancy. The prognosis is poor, particularly when diagnosis is delayed and only incomplete chemotherapy is administered, which is often the case in the low-income settings where eBL is most common [20].

In endemic areas, an eBL diagnosis is usually made on the clinical picture and microscopic examination of cell/tissue morphology of tumour aspirates or biopsies [19]. The pathogenesis of eBL characteristically involves a translocation of the *c-myc* gene from chromosome 8 to the immunoglobulin gene locus on chromosome 14 (heavy chain) (about 80% of cases) or less often to either the κ or the λ light chain locus on chromosome 2 and chromosome 22, respectively [7]. The translocation drives lymphomagenesis by deregulation of proliferation of the affected B-cell clone, due to overexpression of C-MYC protein. Detection of *c-myc* translocation by FISH and detection of C-MYC expression by immunohistochemistry therefore constitute important additional diagnostic assays [19, 21], although these are rarely employed in eBL-endemic settings. In this study, we assessed the sensitivity and specificity of morphologic eBL diagnosis in an endemic setting, and whether diagnostic accuracy could be improved by adding FISH and/or immunohistochemistry.

Retrospective and blinded morphological diagnosis by microscopy of FNA smears by two experts (CS, PHN) at Herlev Hospital, which is a teaching hospital to University of Copenhagen showed good agreement with the original diagnoses made after similar examination of separate smears at the pathology departments at the Ghanaian hospitals admitting the patients in Accra and Kumasi (KBTH and KATH, respectively). Similar assessment of freshly made sections of FFPE tissue blocks stored for up to 8 years yielded a similar picture, although sensitivity and specificity estimates were lower, likely due to problems with inadequate preparation and/or storage of the archival samples.

Confirmation of the original diagnosis by FISH detection of *c-myc* breakage and *c-myc/igh* fusion (*c-myc* translocation) was generally sensitive. Only translocation of *c-myc* to the heavy chain locus was detected. Specificity was fairly low, as we found no evidence of *c-myc* translocation in samples from a substantial proportion of patients originally diagnosed with eBL. These findings were supported by analysis of c-MYC expression by immunohistochemistry. A few patients originally diagnosed with non-eBL showed retrospective evidence of *c-myc* translocation, but this also occurs, albeit less frequently, in other aggressive B-cell lymphomas such as lymphoblastic lymphoma and diffuse large B-cell lymphoma.

It is a weakness of our study that the number of samples available to us was limited, precluding a full retrospective evaluation of the original diagnoses. The poor preservation status of a sizeable proportion of the FFPE biopsies was also a challenge. In addition, we were not able to perform immune-histochemical or -cytochemical confirmation of the haematological or B-cell lineage of the tumour cells, due to the very limited amount of tumour sample material. We could therefore not distinguish mature B-cell lymphomas from other small round blue cell tumours.

## Conclusion

Overall, we conclude that the original diagnoses, which involved laboratory assessment of tumour cell morphology, were reliable, when evaluated by independent retrospective analysis of specimens similar to those available at the time of the original diagnosis. However, diagnostic specificity can probably be improved by introduction of immuno-histochemical analysis for evidence of C-MYC expression. Furthermore, our data support the use of FNA samples for pathology laboratory investigations, in particular when inadequate preservation of biopsy material obtained by surgery makes the justification for this more injurious and complicated procedure questionable.

## Supplementary information

**Additional file 1:** **Table S1.** Comparison of original diagnosis and retrospective assessment by microscopy of Giemsa-stained FNA smears. **Table S2.** Comparison of original diagnosis and retrospective assessment by microscopy of haematoxylin-eosin-stained tissue sections from FFPE tissue blocks. **Table S3.** Comparison of original diagnosis and retrospective assessment by microscopy of FISH-*c-myc*-stained FNA smears. **Table S4.** Comparison of original diagnosis and retrospective assessment by microscopy of FISH-*c-myc-igh*-stained FNA smears. **Table S5.** Comparison of original diagnosis and retrospective assessment by microscopy of FISH-*c-myc*-stained FFPE sections. **Table S6.** Comparison of original diagnosis and retrospective assessment by microscopy of FISH-*c-myc-igh*-stained FFPE sections. **Table S7.** Comparison of original diagnosis and retrospective assessment by microscopy of immunohistochemistry detection of C-MYC expression on FFPE sections.

## Data Availability

All data generated or analysed during this study are included in this published article and its supplementary information files.

## Abbreviations

ABCL: Aggressive B-cell lymphoma
BL: Burkitt lymphoma
eBL: endemic BL
FFPE: Formalin-fixed and paraffin-embedded
FISH: Fluorescence in situ hybridization
FNA: Fine-needle aspirate
KATH: Komfo Anokye Teaching Hospital
KBTH: Korle-Bu Teaching Hospital

## References

[1] Swerdlow SH, Campo E, Harris NL, Jaffe ES, Pileri SA, Stein H, Thiele J (2017). WHO classification of tumours of haematopoietic and lymphoid tissues.

[2] Burkitt D (1958). A sarcoma involving the jaws in African children. Br J Surg.

[3] Burkitt D (1962). A children's cancer dependent on climatic factors. Nature.

[4] Quintana M, Smith-Togobo C, Moormann A, Hviid L. Endemic Burkitt lymphoma - an aggressive childhood cancer linked to *Plasmodium falciparum* exposure, but not to exposure to other malaria parasites. APMIS. (in press). 10.1111/apm.13018 32133709

[5] Nkrumah FK, Perkins IV (1976). Burkitt’s lymphoma: a clinical study of 110 patients. Cancer.

[6] Segbefia CI, Renner LA, Dei-Adomakoh Y, Welbeck J (2013). Changing patterns of childhood cancers at Korle Bu teaching hospital, Accra, Ghana. Postgrad Med J Ghana.

[7] Diebold J, Jaffe ES, Raphael M, Warnke RA, Jaffe ES, Harris NL, Stein H, Vardiman JW (2001). Burkitt lymphoma. Pathology and genetics of tumours of haematopoietic and lymphoid tissues.

[8] Aquino G, Marra L, Curcio MP, De Chiara A, Liguori G, Franco R (2014). Detection of *myc* rearranged by fluorescence *in situ* hybridization FISH: a diagnostic tool. World Cancer Res J.

[9] Buno I, Nava P, Alvarez-Doval A, Alvarez-Rodriguez F, Diez-Martin JL, Menarguez J (2005). Lymphoma associated chromosomal abnormalities can easily be detected by FISH on tissue imprints. An underused diagnostic alternative. J Clin Pathol.

[10] Ogwang MD, Zhao W, Ayers LW, Mbulaiteye SM (2011). Accuracy of Burkitt lymphoma diagnosis in constrained pathology settings: importance to epidemiology. Arch Pathol Lab Med.

[11] Pedersen MO, Gang AO, Clasen-Linde E, Breinholt MF, Knudsen H, Nielsen SL, Poulsen TS, Klausen TW, Hogdall E, Norgaard P (2019). Stratification by MYC expression has prognostic impact in MYC translocated B-cell lymphoma - identifies a subgroup of patients with poor outcome. Eur J Haematol.

[12] Clark Schneider KM, Banks PM, Collie AM, Lanigan CP, Manilich E, Durkin LM, Hill BT, Hsi ED (2016). Dual expression of MYC and BCL2 proteins predicts worse outcomes in diffuse large B-cell lymphoma. Leuk Lymphoma.

[13] Altman DG, Machin D, Bryant TN, Gardner MJ. Statistics with confidence, 2nd edn. London: British Medical Journal; 2000.

[14] Wagener R, Bens S, Toprak UH, Seufert J, Lopez C, Scholz I, Herbrueggen H, Oschlies I, Stilgenbauer S, Schlesner M, et al. Cryptic insertion of MYC exons 2 and 3 into the IGH locus detected by whole genome sequencing in a case of MYC-negative Burkitt lymphoma. Haematologica. 2019. 10.3324/haematol.2018.208140. 10.3324/haematol.2018.208140 PMC7109723 31073073

[15] Tapia G, Lopez R, Munoz-Marmol AM, Mate JL, Sanz C, Marginet R, Navarro JT, Ribera JM, Ariza A (2011). Immunohistochemical detection of MYC protein correlates with MYC gene status in aggressive B cell lymphomas. Histopathology.

[16] Wennborg AD, Altiok E, Moore JP, Ernberg I, Klein G (1991). Differential *c-myc* protein expression in Burkitt's lymphomas and EBV-transformed lymphoblastoid lines. Eur J Cancer.

[17] Nwanze J, Siddiqui MT, Stevens KA, Saxe D, Cohen C (2017). MYC immunohistochemistry predicts *MYC* rearrangements by FISH. Front Oncol.

[18] Burkitt DP (1983). The discovery of Burkitt’s lymphoma. Cancer.

[19] Molyneux EM, Rochford R, Griffin B, Newton R, Jackson G, Menon G, Harrison CJ, Israels T, Bailey S (2012). Burkitt’s lymphoma. Lancet.

[20] Joko-Fru WY, Parkin DM, Borok M, Chokunonga E, Korir A, Nambooze S, Wabinga H, Liu B, Stefan C (2018). Survival from childhood cancers in eastern Africa: a population-based registry study. Int J Cancer.

[21] Dave SS, Fu K, Wright GW, Lam LT, Kluin P, Boerma EJ, Greiner TC, Weisenburger DD, Rosenwald A, Ott G (2006). Molecular diagnosis of Burkitt’s lymphoma. N Engl J Med.

