# Supplementary material for: Reliable cell and tissue morphology-based diagnosis of endemic Burkitt lymphoma in resource-constrained settings in Ghana
Source: BMC Cancer. 2019 Dec 30;19:1270. doi: 10.1186/s12885-019-6488-1 (PMC6937736; doi:10.1186/s12885-019-6488-1)
Supplement: Supplementary file 1 — Additional file 1: Table S1. Comparison of original diagnosis and retrospective assessment by microscopy of Giemsa-stained FNA smears. Table S2. Comparison of original diagnosis and retrospective assessment by microscopy of haematoxylin-eosin-stained tissue sections from FFPE tissue blocks. Table S3. Comparison of original diagnosis and retrospective assessment by microscopy of FISH-c-myc-stained FNA smears. Table S4. Comparison of original diagnosis and retrospective assessment by microscopy of FISH-c-myc-igh-stained FNA smears. Table S5. Comparison of original diagnosis and retrospective assessment by microscopy of FISH-c-myc-stained FFPE sections. Table S6. Comparison of original diagnosis and retrospective assessment by microscopy of FISH-c-myc-igh-stained FFPE sections. Table S7. Comparison of original diagnosis and retrospective assessment by microscopy of immunohistochemistry detection of C-MYC expression on FFPE sections. [file 12885_2019_6488_MOESM1_ESM.docx]

Supplementary material for:

Reliable cell and tissue morphology-based diagnosis of endemic Burkitt lymphoma in resource-constrained settings in Ghana

Cecilia Smith-Togobo,^1,2,3^ Mette Ø Pedersen,^4^ Steffen G Jensen,^4^ Babatunde Duduyemi,^5^ Richard K Gyasi,^6^ Michael F Ofori,^2^ Vivian Paintsil,^7^ Lorna Renner,^8^ Peter Nørgaard^4^ and Lars Hviid^3,9#^

Department of Biochemistry, Cell and Molecular Biology, University of Ghana, Legon, Ghana^1^; Department of Immunology, Noguchi Memorial Institute for Medical Research, University of Ghana, Legon, Ghana^2^; Centre for Medical Parasitology at Department of Immunology and Microbiology, Faculty of Health and Medical Sciences, University of Copenhagen^3^; Department of Pathology, Herlev and Gentofte Hospital, Herlev, Denmark^4^; Department of Pathology, Komfo Anokye Hospital, Kumasi, Ghana^5^; Department of Pathology, Korle‑Bu Teaching Hospital, Accra, Ghana^6^; Department of Child Health, Komfo Anokye Hospital, Kumasi, Ghana^7^; Department of Child Health, Korle‑Bu Teaching Hospital, Accra, Ghana^8^; Department of Infectious Diseases, Rigshospitalet, Copenhagen, Denmark^9^

## Supplementary Table 1. Comparison of original diagnosis and retrospective assessment by microscopy of Giemsa-stained FNA smears

|  |  |  | Retrospective assessment | | | | Validity* | | |
| --- | --- | --- | --- | --- | --- | --- | --- | --- | --- |
|  | Hospital |  | eBL | Non-eBL | Discarded | Total | Sensitivity | Specificity | Accuracy |
| Original diagnosis | KBTH | eBL | 4 | 0 | 0 | 4 | 1.0 [.39 to 1.0] | 1.0 [.29 to 1.0] | 1.0 [.59 to 1.0] |
|  |  | Non-eBL | 0 | 3 | 0 | 3 |  |  |  |
|  |  | All | 4 | 3 | 0 | 7 |  |  |  |
|  | KATH | eBL | 13 | 0 | 0 | 13 | 1.0 [.75 to 1.0] | 1.0 [.63 to 1.0] | 1.0 [.84 to 1.0] |
|  |  | Non-eBL | 0 | 8 | 0 | 8 |  |  |  |
|  |  | All | 13 | 8 | 0 | 21 |  |  |  |
|  | All | eBL | 17 | 0 | 0 | 17 | 1.0 [.80 to 1.0] | 1.0 [.72 to 1.0] | 1.0 [.88 to 1.0] |
|  |  | Non-eBL | 0 | 11 | 0 | 11 |  |  |  |
|  |  | All | 17 | 11 | 0 | 28 |  |  |  |

*95% confidence intervals for estimates are shown in square brackets.

## Supplementary Table 2. Comparison of original diagnosis and retrospective assessment by microscopy of haematoxylin-eosin-stained tissue sections from FFPE tissue blocks

|  |  |  | Retrospective assessment | | | | Validity* | | |
| --- | --- | --- | --- | --- | --- | --- | --- | --- | --- |
|  | Hospital |  | eBL | Non-eBL | Discarded | Total | Sensitivity | Specificity | Accuracy |
| Original diagnosis | KBTH | eBL | 21 | 3 | 11 | 35 | 0.84 [.64 to .95] | 0.90 [.74 to .98] | .88 [.76 to .95] |
|  |  | Non-eBL | 4 | 28 | 5 | 37 |  |  |  |
|  |  | All | 25 | 31 | 16 | 72 |  |  |  |
|  | KATH | eBL | 14 | 4 | 2 | 20 | 0.82 [.57 to .96] | 0.67 [.35 to .90] | 0.76 [.56 to .90] |
|  |  | Non-eBL | 3 | 8 | 8 | 19 |  |  |  |
|  |  | All | 17 | 12 | 10 | 39 |  |  |  |
|  | All | eBL | 35 | 7 | 13 | 55 | 0.83 [.69 to .93] | 0.84 [.69 to .93] | 0.84 [.74 to .91] |
|  |  | Non-eBL | 7 | 36 | 13 | 56 |  |  |  |
|  |  | All | 42 | 43 | 26 | 111 |  |  |  |

*95% confidence intervals for estimates are shown in square brackets.

## Supplementary Table 3. Comparison of original diagnosis and retrospective assessment by microscopy of FISH-c‑myc‑stained FNA smears

|  |  |  | Retrospective assessment | | | | Validity* | | |
| --- | --- | --- | --- | --- | --- | --- | --- | --- | --- |
|  | Hospital |  | eBL | Non-eBL | Discarded | Total | Sensitivity | Specificity | Accuracy |
| Original diagnosis | KBTH | eBL | 2 | 0 | 0 | 2 | 0.67 [.09 to .99] | n.a. | 0.67 [.09 to .99] |
|  |  | Non-eBL | 1 | 0 | 0 | 1 |  |  |  |
|  |  | All | 3 | 0 | 0 | 3 |  |  |  |
|  | KATH | eBL | 10 | 1 | 0 | 11 | 0.83 [.52 to .98] | 0.80 [.28 to .99] | 0.82 [.57 to .96] |
|  |  | Non-eBL | 2 | 4 | 0 | 6 |  |  |  |
|  |  | All | 12 | 5 | 0 | 17 |  |  |  |
|  | All | eBL | 12 | 1 | 0 | 13 | 0.80 [.52 to .96] | 0.80 [.28 to .99] | 0.80 [.56 to .94] |
|  |  | Non-eBL | 3 | 4 | 0 | 7 |  |  |  |
|  |  | All | 15 | 5 | 0 | 20 |  |  |  |

*95% confidence intervals for estimates are shown in square brackets.

## Supplementary Table 4. Comparison of original diagnosis and retrospective assessment by microscopy of FISH-c‑myc-igh‑stained FNA smears

|  |  |  | Retrospective assessment | | | | Validity* | | |
| --- | --- | --- | --- | --- | --- | --- | --- | --- | --- |
|  | Hospital |  | eBL | Non-eBL | Discarded | Total | Sensitivity | Specificity | Accuracy |
| Original diagnosis | KBTH | eBL | 2 | 0 | 0 | 2 | 0.67 [.09 to .99] | n.a. | 0.67 [.09 to .99] |
|  |  | Non-eBL | 1 | 0 | 0 | 1 |  |  |  |
|  |  | All | 3 | 0 | 0 | 3 |  |  |  |
|  | KATH | eBL | 9 | 2 | 0 | 11 | 0.75 [.43 to .95] | 0.60 [.15 to .95] | 0.71 [.44 to .90] |
|  |  | Non-eBL | 3 | 3 | 0 | 6 |  |  |  |
|  |  | All | 12 | 5 | 0 | 17 |  |  |  |
|  | All | eBL | 11 | 2 | 0 | 13 | 0.73 [.45 to .92] | 0.60 [.15 to .95] | 0.70 [.46 to .88] |
|  |  | Non-eBL | 4 | 3 | 7 | 0 |  |  |  |
|  |  | All | 15 | 5 | 0 | 20 |  |  |  |

*95% confidence intervals for estimates are shown in square brackets.

## Supplementary Table 5. Comparison of original diagnosis and retrospective assessment by microscopy of FISH-c‑myc‑stained FFPE sections

|  |  |  | Retrospective assessment | | | | Validity* | | |
| --- | --- | --- | --- | --- | --- | --- | --- | --- | --- |
|  | Hospital |  | eBL | Non-eBL | Discarded | Total | Sensitivity | Specificity | Accuracy |
| Original diagnosis | KBTH | eBL | 5 | 3 | 4 | 12 | 0.83 [.36 to 1.0] | 0.67 [.30 to .93] | 0.73 [.45 to .92] |
|  |  | Non-eBL | 1 | 6 | 1 | 8 |  |  |  |
|  |  | All | 6 | 9 | 5 | 20 |  |  |  |
|  | KATH | eBL | 2 | 4 | 4 | 10 | 1.0 [.16 to 1.0] | 0.0 [.00 to .60] | 0.33 [.04 to .78] |
|  |  | Non-eBL | 0 | 0 | 0 | 0 |  |  |  |
|  |  | All | 2 | 4 | 4 | 10 |  |  |  |
|  | All | eBL | 7 | 7 | 8 | 22 | 0.88 [.47 to 1.0] | 0.46 [.19 to .75] | 0.62 [.38 to .82] |
|  |  | Non-eBL | 1 | 6 | 1 | 8 |  |  |  |
|  |  | All | 8 | 13 | 9 | 30 |  |  |  |

*95% confidence intervals for estimates are shown in square brackets.

## Supplementary Table 6. Comparison of original diagnosis and retrospective assessment by microscopy of FISH-c‑myc-igh‑stained FFPE sections

|  |  |  | Retrospective assessment | | | | Validity* | | |
| --- | --- | --- | --- | --- | --- | --- | --- | --- | --- |
|  | Hospital |  | eBL | Non-eBL | Discarded | Total | Sensitivity | Specificity | Accuracy |
| Original diagnosis | KBTH | eBL | 19 | 10 | 5 | 34 | 0.95 [.75 to 1.0] | 0.52 [.30 to .74] | 0.73 [.57 to .86] |
|  |  | Non-eBL | 1 | 11 | 6 | 18 |  |  |  |
|  |  | All | 20 | 21 | 11 | 52 |  |  |  |
|  | KATH | eBL | 4 | 4 | 9 | 17 | 1.0 [.40 to 1.0] | 0.0 [0.0 to .60] | 0.50 [.16 to .84] |
|  |  | Non-eBL | 0 | 0 | 2 | 2 |  |  |  |
|  |  | All | 4 | 4 | 11 | 19 |  |  |  |
|  | All | eBL | 23 | 14 | 14 | 51 | 0.96 [.79 to 1.0] | 0.44 [.24 to .65] | 0.69 [.55 to .82] |
|  |  | Non-eBL | 1 | 11 | 8 | 20 |  |  |  |
|  |  | All | 24 | 25 | 22 | 71 |  |  |  |

*95% confidence intervals for estimates are shown in square brackets.

## Supplementary Table 7. Comparison of original diagnosis and retrospective assessment by microscopy of immunohistochemistry detection of C‑MYC expression on FFPE sections

|  |  |  | Retrospective assessment | | | | Validity* | | |
| --- | --- | --- | --- | --- | --- | --- | --- | --- | --- |
|  | Hospital |  | eBL | Non-eBL | Discarded | Total | Sensitivity | Specificity | Accuracy |
| Original diagnosis | KBTH | eBL | 15 | 6 | 0 | 21 | 0.79 [.54 to .94] | 0.45 [.17 to .77] | 0.67 [.47 to .83] |
|  |  | Non-eBL | 4 | 5 | 0 | 9 |  |  |  |
|  |  | All | 19 | 11 | 0 | 30 |  |  |  |
|  | KATH | eBL | 11 | 2 | 1 | 14 | 0.79 [.49 to .95] | 0.33 [.01 to .91] | 0.71 [.44 to .90] |
|  |  | Non-eBL | 3 | 1 | 0 | 4 |  |  |  |
|  |  | All | 14 | 3 | 1 | 18 |  |  |  |
|  | All | eBL | 26 | 8 | 1 | 35 | 0.79 [.61 to .91] | 0.43 [.18 to .71] | 0.68 [.53 to .81] |
|  |  | Non-eBL | 7 | 6 | 0 | 13 |  |  |  |
|  |  | All | 33 | 14 | 1 | 48 |  |  |  |

*95% confidence intervals for estimates are shown in square brackets.
